# Alterations of the intestinal microbiota in age-related macular degeneration

**DOI:** 10.3389/fmicb.2023.1069325

**Published:** 2023-04-05

**Authors:** Yuanyuan Zhang, Tianyu Wang, Zhongqi Wan, Jianhao Bai, Yawen Xue, Rushun Dai, Minli Wang, Qing Peng

**Affiliations:** ^1^Department of Ophthalmology, Shanghai Tenth People’s Hospital, Tongji University School of Medicine, Shanghai, China; ^2^Department of Clinical Laboratory Medicine, Shanghai Tenth People's Hospital, Tongji University School of Medicine, Shanghai, China

**Keywords:** age-related macular degeneration, intestinal microbiota, gut-retina axis, microbial diversity, metabolic pathway

## Abstract

**Purpose:**

Age-related macular degeneration (AMD) is the leading cause of vision loss in those over the age of 50. Recently, intestinal microbiota has been reported to be involved in the pathogenesis of ocular diseases. The purpose of this study was to discover more about the involvement of the intestinal microbiota in AMD patients.

**Methods:**

Fecal samples from 30 patients with AMD (AMD group) and 17 age- and sex-matched healthy controls (control group) without any fundus disease were collected. DNA extraction, PCR amplification, and 16S rRNA gene sequencing of the samples were performed to identify intestinal microbial alterations. Further, we used BugBase for phenotypic prediction and PICRUSt2 for KEGG Orthology (KO) as well as metabolic feature prediction.

**Results:**

The intestinal microbiota was found to be significantly altered in the AMD group. The AMD group had a significantly lower level of *Firmicutes* and relatively higher levels of *Proteobacteria* and *Bacteroidota* compared to those in the control group. At the genus level, the AMD patient group showed a considerably higher proportion of *Escherichia-Shigella* and lower proportions of *Blautia* and *Anaerostipes* compared with those in the control group. Phenotypic prediction revealed obvious differences in the four phenotypes between the two groups. PICRUSt2 analysis revealed KOs and pathways associated with altered intestinal microbiota. The abundance of the top eight KOs in the AMD group was higher than that in the control group. These KOs were mainly involved in lipopolysaccharide biosynthesis.

**Conclusion:**

The findings of this study indicated that AMD patients had different gut microbiota compared with healthy controls, and that AMD pathophysiology might be linked to changes in gut-related metabolic pathways. Therefore, intestinal microbiota might serve as non-invasive indicators for AMD clinical diagnosis and possibly also as AMD treatment targets.

## Introduction

Age-related macular degeneration (AMD) is a multifactorial disorder having risk factors, such as heredity, environment (smoking, light, diet, etc.), age, and race (Mitchell et al., 2018). AMD mainly affects the macular region of the retina. In severe cases, it can lead to vision loss and even blindness. Wet AMD is an advanced stage of AMD that is mainly characterized by choroidal neovascularization (CNV), macular hemorrhage, exudation, and edema. Current clinical diagnosis of AMD is done through funduscopy, fluorescein angiography, optical coherence tomography angiography, and, occasionally, indocyanine green angiography (Told et al., 2018). The first-line treatment for advanced AMD is an intravitreal injection of anti-vascular endothelial growth factor drugs; however, some patients may experience disease relapse after operation and even show no response to treatment. Therefore, AMD pathogenesis needs further exploration. Recently, some researchers have postulated that the intestinal microbiota may be involved in AMD pathogenesis, providing a new avenue for exploring the etiology and therapeutic modalities for AMD (Andriessen et al., 2016; Zinkernagel et al., 2017; Zysset-Burri et al., 2020).

The gut microbiota is composed of bacteria, archaea, viruses, fungi, and eukaryotic microbes. Among these, bacteria are widely studied by researchers. The most common bacterial phyla are *Firmicutes*, *Bacteroidota*, *Actinobacteria*, and *Proteobacteria* (Lynch and Pedersen, 2016). The intestinal microbiota interacts with the host to maintain the normal functioning of the human body. It plays many roles in immunological regulation, metabolism, and intercellular communication to contribute to the homeostasis of the body (Jandhyala et al., 2015). However, intestinal microbiota abnormalities have been linked to disorders ranging from localized gastroenterology to systemic diseases (Hou et al., 2022). The view that the gut microbiota affects anatomically distant organs is very confusing, especially the regulatory effect of the gut microbiota on immune-privileged sites, like the eyes (Zárate-Bladés et al., 2017). Over the past few years, groundbreaking research on the relationship between intestinal microbiota and the retina (gut-retina axis) has increased (Lin, 2019; Scuderi et al., 2021).

Disturbances in the intestinal microbiota and its metabolites may cause systemic inflammation by stimulating both the innate and adaptive responses of the immune system. This inflammation may be the reason for many diseases, including the ocular diseases, as revealed by human and animal studies (Floyd and Grant, 2020). Ten percent of individuals with inflammatory bowel disease develop eye diseases, such as episcleritis, uveitis, and conjunctivitis (Vavricka et al., 2015). One study (Astafurov et al., 2014) found that low levels of lipopolysaccharide (LPS) administered subcutaneously to induce chronic inflammation in two animal models of glaucoma could increase optic nerve axon loss and expression of Toll-like receptor (TLR) and complement system pathway genes, which could cause microglial activation in the affected tissue. Moreover, microglia participate in the pathology of glaucomatous and other neurodegenerative diseases and express high levels of TLR4 (Amor et al., 2010; Ebneter et al., 2010). Studies in humans have identified an association between TLR4 polymorphisms and normal tension glaucoma (Shibuya et al., 2008). Administrating a TLR4 inhibitor, naloxone, orally was found to protect retinal ganglion cells and optic nerve axons against damage (Astafurov et al., 2014). It is very likely that an increase in the level of LPS in circulation plays a vital role in inducing diabetes-related inflammation. Microbial metabolites and components migrate into the circulation through the damaged intestinal barrier; this could trigger oxidative stress and pro-inflammatory pathways and ultimately lead to vascular dysfunction (Burcelin, 2016; Xu and Chen, 2017). One mechanism that possibly links the imbalance of the gut microbiota to diabetic retinopathy may involve the activation of TLR, the key mediator of innate immunity, to cause systemic inflammatory and abnormal vascular responses (Li et al., 2017). Fernandes et al. found that hyperglycemia was related to an altered intestinal barrier, causing the loss of immune system homeostasis in association with systemic chronic inflammation and even involving retinal vessels. They hypothesized that all these changes contributed to retinal inflammation, breakdown of the blood-retinal barrier, apoptosis, and neovascularization, leading to diabetic retinopathy progression (Fernandes et al., 2019). The combination of LPS and TLR4 was found to trigger a series of reactions, eventually leading to the release of pro-inflammatory molecules, which interfere with glucose and insulin metabolism (Blandino et al., 2016). A study performed on type 1 diabetes patients with and without diabetic retinopathy demonstrated that they all had higher levels of LPS compared to individuals with normal glucose tolerance (Fernandes et al., 2019). A growing number of studies have also shown that type 2 diabetes is associated with an alteration of the intestinal microbiota (Qin et al., 2012; Karlsson et al., 2013). Potential mechanisms underlying this association include increased inflammation, oxidative stress, and vascular permeability (Allin et al., 2015; Arora and Bäckhed, 2016). A study of a transgenic mouse model of uveitis, showed that the intestinal microbiota could activate autoreactive T cells that produced IL-17 and thereby directly triggered uveitis (Horai et al., 2015). The reason behind this direct association between the gut and the eye is that auto-pathogenic T cells in the retina receive a microbiota-dependent activation signal through their clonotypic T cell receptor in the gut (Horai et al., 2015). Another study revealed, using different uveitis animal models, that administration of broad-spectrum antibiotics increased regulatory T cells in the lamina propria and decreased effector CD4+ T cells (Nakamura et al., 2016). In humans, uveitis and related autoimmune diseases are related to human leukocyte antigen-B27, which is known to be associated with intestinal disorders in human and animal models that cause increased intestinal permeability and innate and adaptive immune responses (Nakamura et al., 2016). Although a growing number of studies have found important roles played by intestinal microbiota in many diseases, these roles need to be explored further.

Recent studies employed CNV models to investigate the involvement of intestinal microbiota in the pathogenesis of AMD (Andriessen et al., 2016). These studies explored indirect effects of dietary changes on the retina mediated by the composition of the gut microbiota. However, the pathway underlying the direct involvement of intestinal microbiota in AMD remains to be explored. There have only been a few studies to date on changes in the intestinal microbiota of patients with AMD (Zinkernagel et al., 2017; Zysset-Burri et al., 2020). Moreover, intestinal microbiota vary with geographical location (Vaiserman et al., 2017). As a result, more investigations and validations at other research sites are required to ascertain the effects of changes in gut microbiota on AMD patients. In the present study, to elucidate the mechanisms underlying AMD pathogenesis, we employed 16S rRNA gene sequencing to describe the intestinal microbiota of AMD patients and forecast probable metabolic pathways they may affect. Through this method, we searched for potential microbial biomarkers for the development of AMD and innovative treatment methods for AMD.

## Materials and methods

### Study participants

This study was approved by the ethics committee of Shanghai Tenth People’s Hospital (Chinese Clinical Trial Registry approval number: ChiCTR2100051816, https://www.chictr.org.cn/) and was in accordance with the Declaration of Helsinki. Before the subjects were enrolled in the study, they all signed informed consent. The inclusion criteria were as follows: patients above 50 years old, living in Shanghai and having advanced AMD clinical features after receiving an ophthalmic examination, which includes visual acuity examination, dilated fundoscopy, fundus color photography, and optical coherence tomography angiography. Age and sex of the healthy controls were matched with the patients, and no abnormality was found in the fundus. The exclusion criteria were as follows: antibiotics-use within 3 months before enrollment; a history of gastrointestinal surgery in the last 5 years; serious mental, neurological, cardiovascular, respiratory diseases, or diseases of other systems.

### Sample collection and DNA extraction

Fecal samples were collected and promptly frozen at −80°C prior to DNA extraction. The E.Z.N.A.® soil DNA Kit (Omega Bio-Tek, Norcross, GA, United States) was used to extract microbial DNA from 47 fecal samples according to the manufacturer’s instructions. The DNA extract was checked on 1% agarose gel. The concentration and purity of bacterial DNA were determined by the NanoDrop 2000 UV–vis spectrophotometer (Thermo Scientific, Wilmington, United States).

### PCR and Illumina MiSeq sequencing

The V3-V4 variable region of the 16S rRNA gene was amplified by PCR using the primer pairs 338F (5′-ACTCCTACGGGAGGCAGCAG-3′) and 806R (5′-GGACTACHVGGGTWTCTAAT-3′) by an ABI GeneAmp® 9700 PCR thermocycler (ABI, CA, United States). The PCR reactions were performed as follows: initial denaturation at 95°C for 3 min, followed by 27 cycles of denaturing at 95°C for 30 s, annealing at 55°C for 30 s and extension at 72°C for 45 s, and single extension at 72°C for 10 min, and end at 4°C. The PCR mixtures contain 5 × TransStart FastPfu buffer 4 μL, 2.5 mM dNTPs 2 μL, forward primer (5 μM) 0.8 μL, reverse primer (5 μM) 0.8 μL, TransStart FastPfu DNA Polymerase 0.4 μL, template DNA 10 ng, and finally ddH2O up to 20 μL. PCR reactions were performed in triplicate. The amplicon products were extracted from 2% agarose gel and purified using the AxyPrep DNA Gel Extraction Kit (Axygen Biosciences, Union City, CA, United States) according to manufacturer’s instructions and quantified using Quantus™ Fluorometer (Promega, United States).

Then, the amplicons were pooled in equimolar and sequenced using the Illumina MiSeq platform (Illumina, San Diego, United States) according to the standard protocols by Majorbio Bio-Pharm Technology Co. Ltd. (Shanghai, China).

### Processing of sequencing data

Paired-end 16S rRNA gene sequencing data were demultiplexed, quality-filtered using Trimmomatic software, and merged using FLASH v1.2.11 software.^1^ The detailed criteria are as follows: (i) The 300-bp reads were truncated at any site receiving an average quality score of less than 20 over a 50-bp sliding window, and truncated reads that were shorter than 50-bp and contained ambiguous characters were discarded; (ii) Only overlapping sequences >10-bp were assembled and the maximum mismatch ratio of overlap region is 0.2. Reads that could not be assembled were discarded; and (iii) Based on the barcode and primers, samples were distinguished, and the sequence direction was adjusted.

UPARSE (version 7.1, http://drive5.com/uparse/) was used to cluster high-quality sequences into operational taxonomic units (OTUs), using 97% similarity as a cutoff. The taxonomy of each OTU representative sequence was analyzed by the Ribosomal Database Project Classifier^2^ against the 16S rRNA database (Silva SSU138, https://www.arb-silva.de/) using a confidence threshold of 0.7.

### Functional prediction

BugBase^3^ is a kind of microbial analysis tools, which can determine high levels of phenotypes in the samples and perform phenotypic prediction. PICRUSt2^4^ is a software package for functional prediction of 16S rRNA amplicon sequencing results. We used PICRUSt2 for KO and pathway prediction.

### Statistical analysis

Mothur (version v.1.30.2, https://www.mothur.org/wiki/Download_mothur) was used to analyze alpha diversity based on the OTU level. The difference was considered statistically significant when *p* < 0.05 and the average abundance level of the species was >1%. QIIME (v1.9.1, http://qiime.org/install/index.html) was used to quantify beta diversity for the samples based on rarefied OTU counts. Data were further analyzed using R software (version 3.3.1). Partial least squares discriminant analysis (PLS-DA) was performed to explore the difference among the microbial communities in both groups using “mixOmics” package. The linear discriminant analysis (LDA) effect size (LEfSe) was used to identify different bacterial taxa between AMD and healthy individuals. Non-parametric Wilcoxon rank-sum test was used to analyze the significant difference between two groups of sample species. Student’s *t*-test was used to compare continuous variables and chi-square test was used to analyze categorical variables using SPSS 25.0 (IBM Corporation, Armonk, NY, United States). The significant difference of KO and metabolic pathway between the two groups was analyzed by STAMP (Parks et al., 2014).

## Results

### Baseline characteristics of the study population

We recruited 30 AMD patients and 17 healthy controls to participate in the present study. There was no significant difference in gender and age between the two groups. The clinical characteristics of body mass index (BMI), hypertension, hyperglycemia, drinking, and smoking history were also comparable between the AMD group and control group. The baseline characteristics of study participants are shown in Table 1.

**Table 1:** Clinical features of AMD patients and controls in this study.

	AMD (*n* = 30)	Control (*n* = 17)	*p* value
Age (year)	69.87 ± 6.70	66.59 ± 7.32	0.126
Sex, male (*n*, %)	16 (53%)	8 (47%)	0.679
BMI (kg/m^2^)	23.73 ± 2.85	24.11 ± 3.01	0.666
Drinking history (*n*, %)	5 (17%)	2 (12%)	1.000
Smoking (*n*, %)	3 (10%)	3 (18%)	0.653
Hypertension (*n*, %)	16 (53%)	5 (29%)	0.113
Diabetes (*n*, %)	6 (20%)	2 (12%)	0.692

AMD, age-related macular degeneration; BMI, body mass index.

### Fecal bacterial diversity in patients with AMD

**Figure 1:**
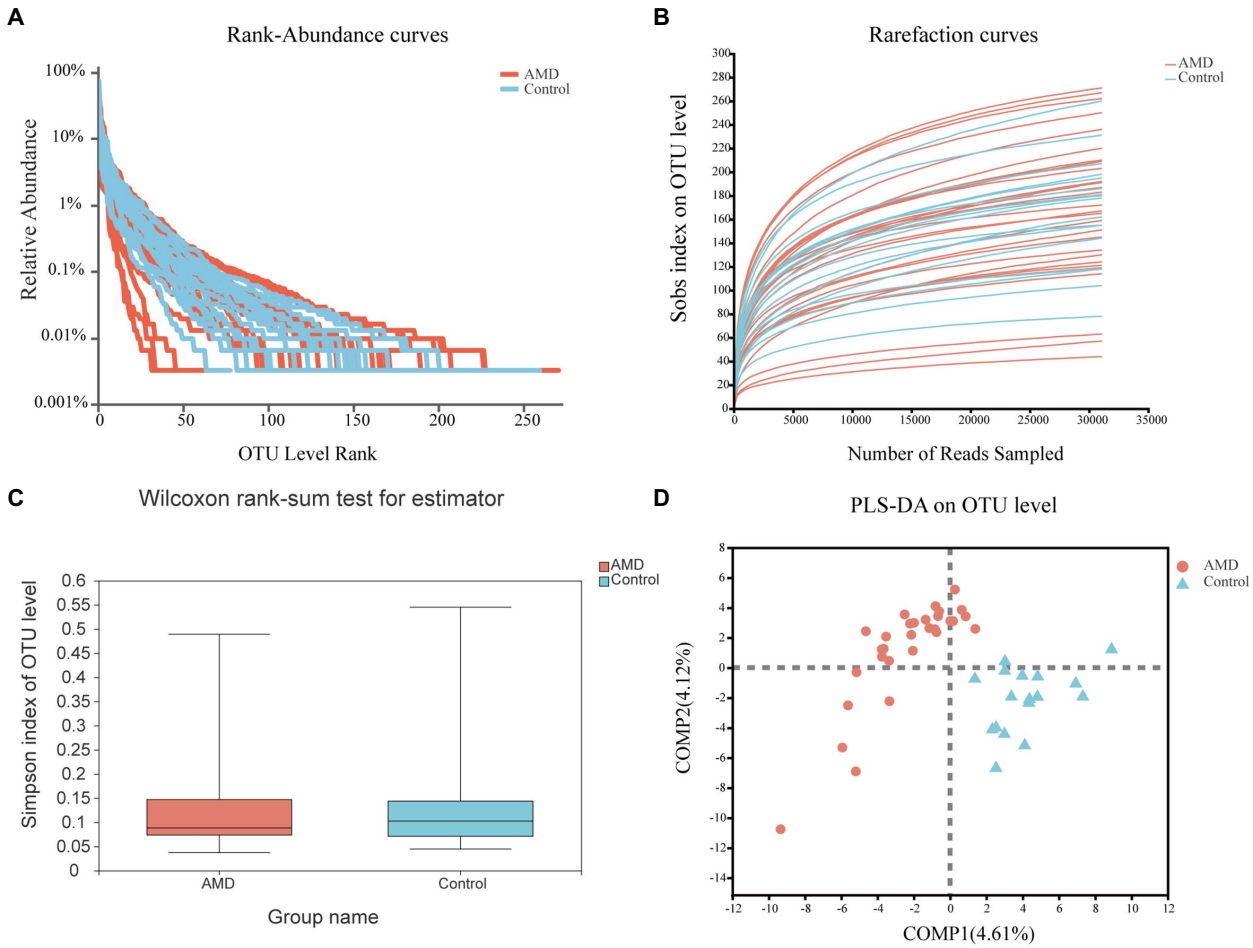
General sequencing characteristics of the 47 fecal samples and comparison of diversity in the age-related macular degeneration (AMD) patients and controls at the OUT level. **(A)** Rank-abundance curves of the gut microbiota. **(B)** Rarefaction curves (Sobs index) of the gut microbiota. **(C)** Comparison of alpha diversity in the AMD patients and controls. The Simpson index reflected the alpha diversity. **(D)** PLS-DA of the microbiome.

High-throughput sequencing results of the microbial 16S rRNA V3–V4 hypervariable regions revealed abundant microbial taxa in the samples. A random rarefaction of sample reads according to the smallest read length was first performed for further analysis. Rank-abundance curves at the OTU level showed that the species were distributed evenly and abundantly (Figure 1A). The four most common phyla were *Firmicutes*, *Bacteroidota*, *Proteobacteria*, and *Actinobacteria*. Rarefaction curves showed that, as the sample size increased, the curves tended to be flat, which demonstrated that the sequencing depth of samples covered almost all species (Figure 1B). The abovementioned results showed that the sequencing data were sufficient, and no more OTUs could be found by acquiring more sequencing data. In the alpha diversity analysis, based on the OTU level, the Simpson index in the fecal samples of AMD patients was found to be lower than that in the fecal samples of healthy individuals (Figure 1C). The Chao1, Observed-species, and Shannon indexes of intestinal microbiota also differed between the two groups (Supplementary Figure 1). In the beta diversity analysis, PLS-DA showed a clear difference in samples between the two groups, suggesting a difference between their gut bacterial compositions (Figure 1D).

### Fecal bacterial composition in patients with AMD

**Figure 2:**
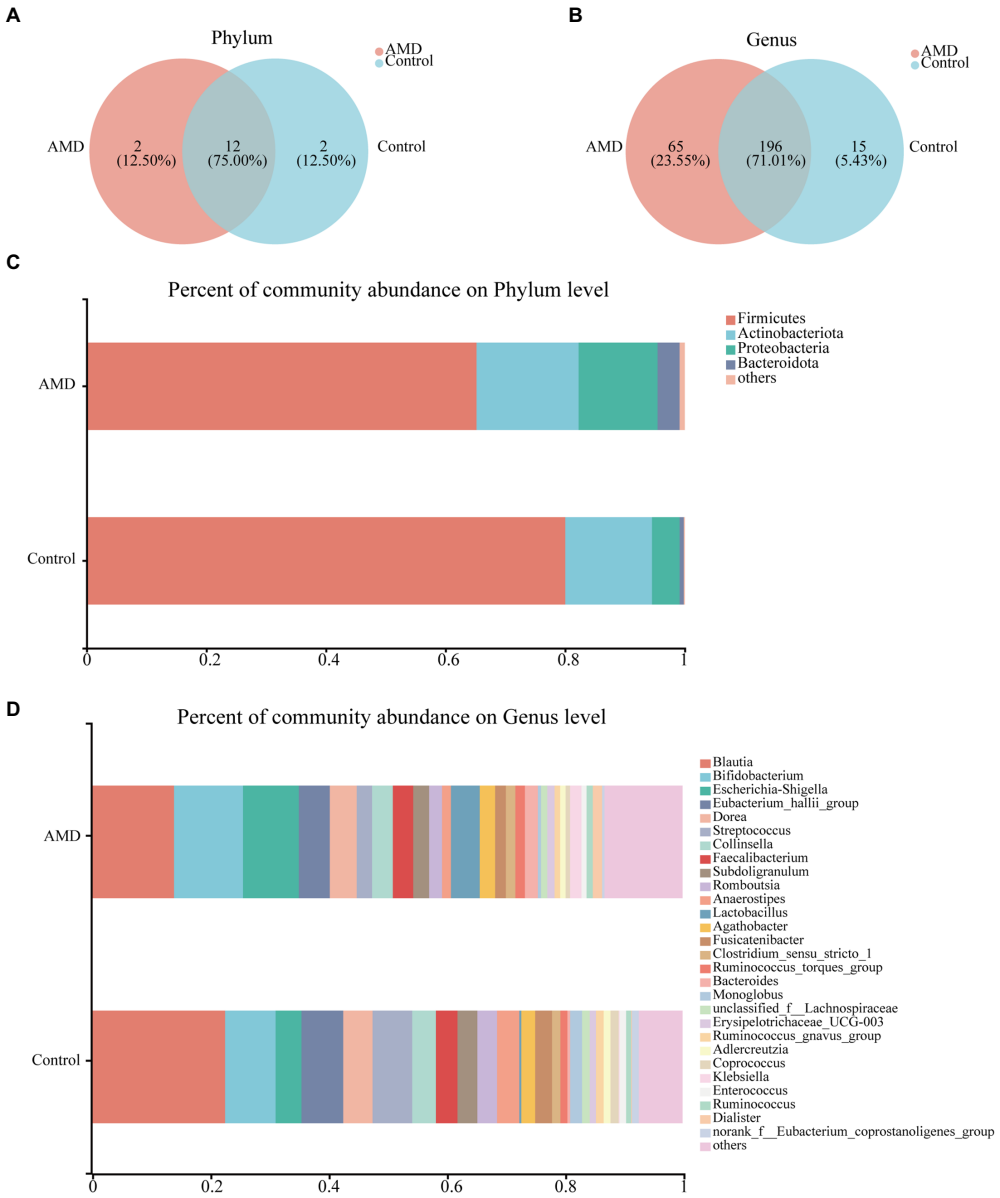
Fecal bacterial composition in patients with AMD and controls. **(A,B)** The species Venn diagram at the phylum and genus level, overlapping parts indicated common species. **(C)** Taxonomic distributions of bacteria at the phylum level in the AMD patients and controls. **(D)** Taxonomic distributions of bacteria at the genus level in the AMD patients and controls.

To investigate the gut microbiota signature, we assessed the relative abundances of bacteria at the phylum and genus levels. In total, 16 phyla and 276 genera were identified, and 12 of the 16 phyla were shared between the AMD and healthy groups. *Deinococcota* and *WPS-2* were unique to the AMD group, whereas *Acidobacteriota* and *Planctomycetota* were specific to the control group (Figure 2A). A total of 196 genera were found in the two groups; however, 65 genera were specific to the AMD group and 15 genera were only present in the control group (Figure 2B). At the phylum level, *Firmicutes*, *Actinobacteria*, *Proteobacteria*, and *Bacteroidota* were the predominant phyla in both AMD and control groups (Figure 2C). Both groups had *Blautia* in the highest proportion at the genus level. At the same time, the abundance of *Blautia* was lower in the AMD group than in the control group (Figure 2D, Supplementary Figure 2A). Overall, the intestinal bacterial compositions in the AMD group were altered at different taxonomic levels when compared to those in the control group.

### Statistical analysis of taxonomic differences between AMD and control groups

**Figure 3:**
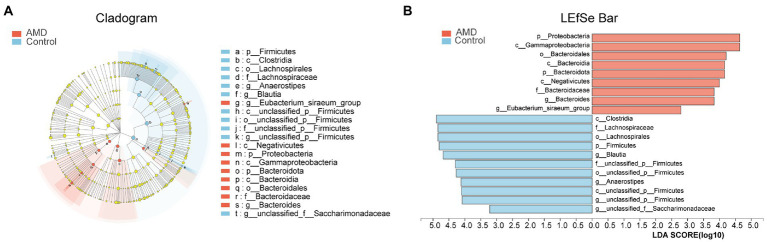
Fecal microbiota profile in the both groups at different taxonomic levels. **(A)** Cladogram of the linear discriminant analysis (LDA) effect size (LEfSe) analysis from the phylum level to the genus level of the microbiota in the AMD patients and controls. **(B)** Bar graph of LDA scores to screen: altered bacteria from phylum level to genus level (LDA score > 2.5).

The structures of intestinal microbiota in the two groups were analyzed at different taxonomic levels. As shown in Figure 3A, a cladogram of the linear discriminant analysis (LDA) effect size (LEfSe) from the phylum level to the genus level revealed 20 taxa that were significantly different between the two groups. According to the LDA scores, *Firmicutes* were less abundant in the AMD group than in the control group. In contrast, the abundances of *Proteobacteria* and *Bacteroidota* were higher in the AMD group compared with those in the control group. At the genus level, the abundances of *Blautia* and *Anaerostipes* were much lower in the AMD patients compared with those in the healthy controls. The abundances of *Bacteroides* and *Eubacterium_siraeum_group* in the AMD group were higher than those in the control group (LDA > 2.5, Figure 3B; Supplementary Figure 2).

**Figure 4:**
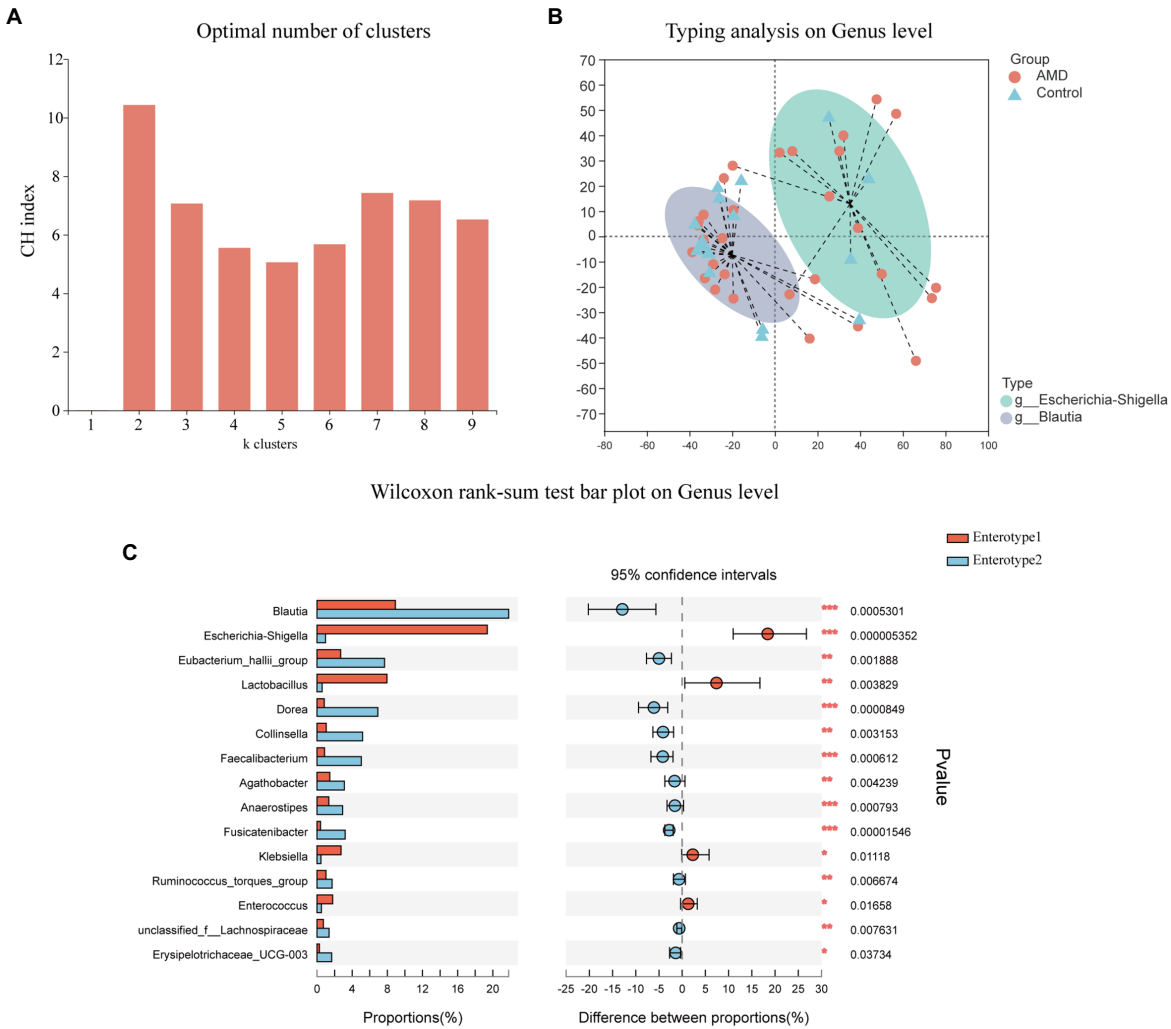
Enterotypes of the intestinal microbiome in AMD patients and healthy controls using Jensen-Shannon distance. **(A)** The optimal number of enterotypes was two as indicated by the maximum CH index at two clusters. **(B)** The figure showed the clustering on the first two principal components. Patients (*n* = 30) and controls (*n* = 17) were denoted by circles and triangles, respectively. **(C)** Comparison between the two enterotypes at the genus level.

We next employed the PAM algorithm with Jensen-Shannon distance to cluster both groups into enterotypes at the genus level (Figure 4). The optimal number of clusters was two according to the Calinski-Harabasz (CH) index (Figure 4A). As shown in Figures 4B,C, the abundance of *Escherichia-Shigella* was relatively high among and representative of enterotype 1 genera; similarly, *Blautia* was the most abundant and representative among enterotype 2 genera. The above results further showed that the AMD group had a large proportion of *Escherichia-Shigella* and the control group had a large proportion of *Blautia*.

### Functional predictive analysis

**Figure 5:**
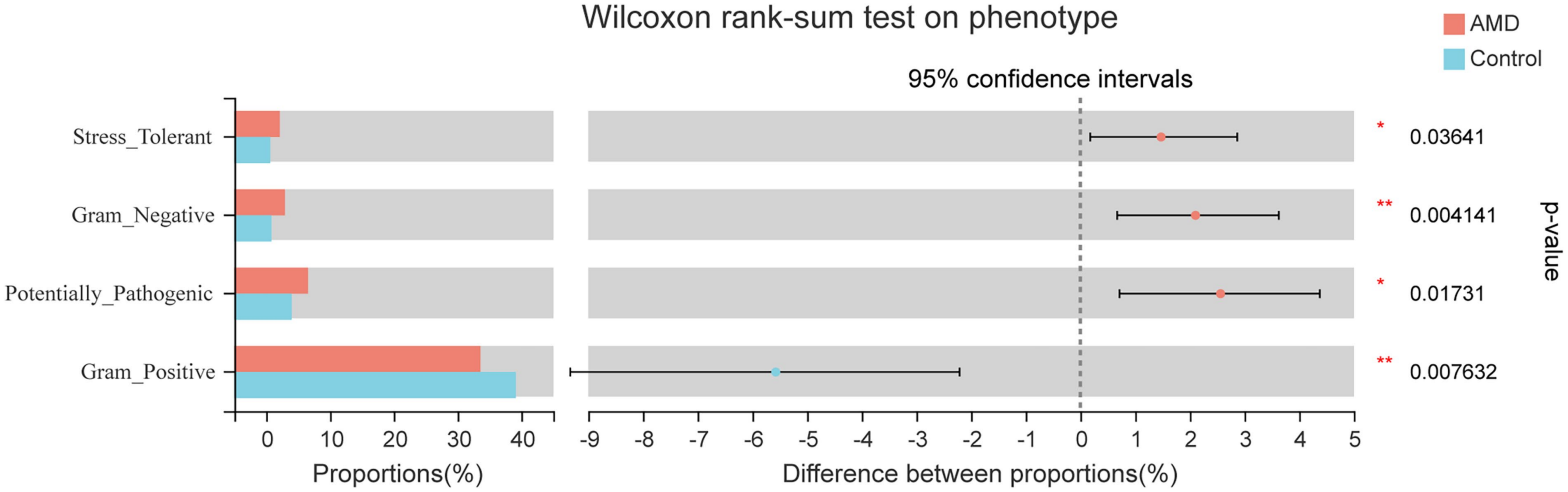
Phenotypic prediction difference test between AMD patients and controls.

**Figure 6:**
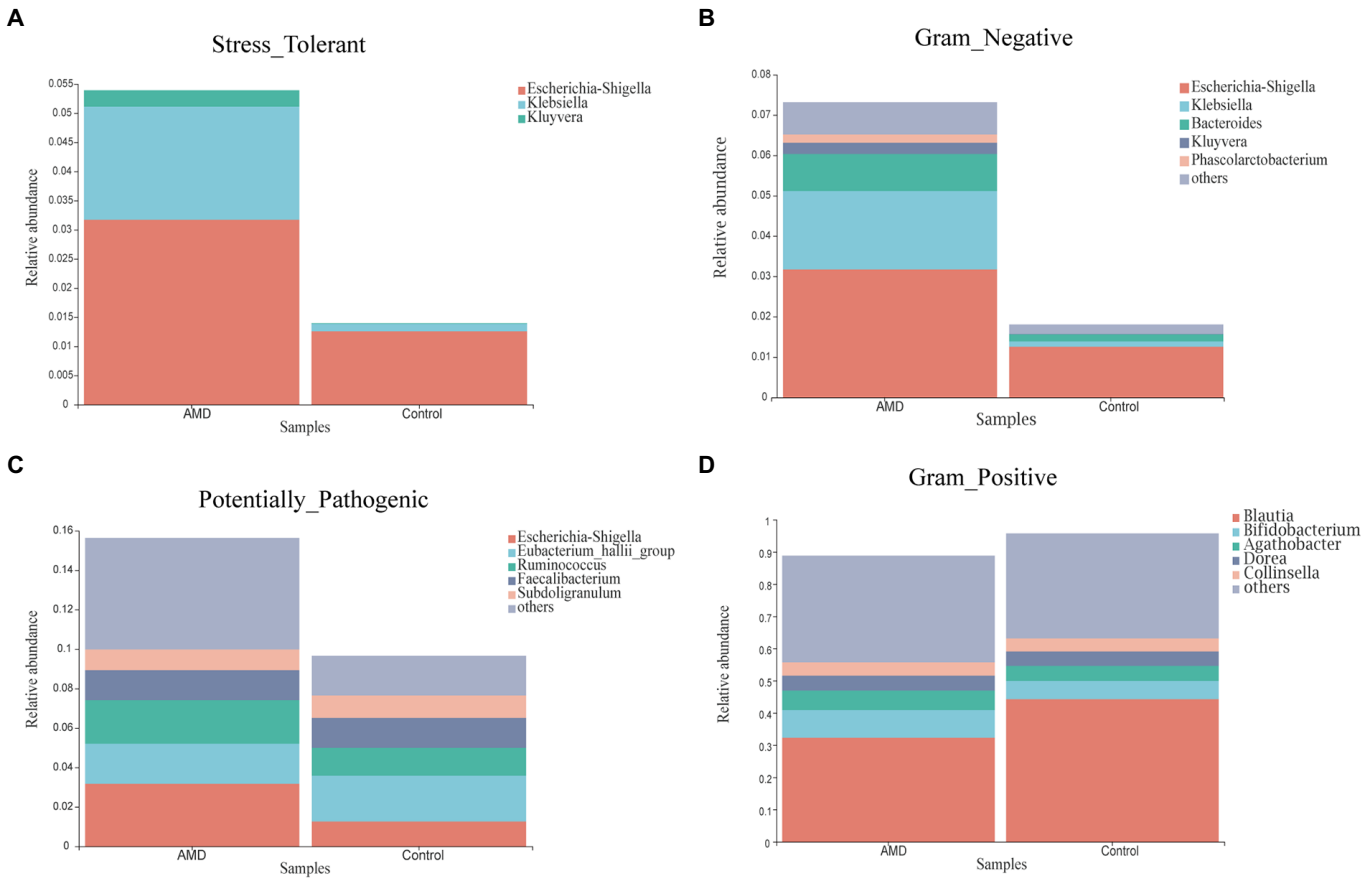
The relative abundance of microbial phenotypes in patients with AMD and controls. **(A)** Stress-Tolerant. **(B)** Gram-Negative. **(C)** Potentially-Pathogenic. **(D)** Gram-Positive.

Phenotypic prediction was performed using BugBase, and four phenotypes were found to have an obvious difference between the two groups (Figure 5). The relative abundances of microorganisms with Stress-Tolerant, Gram-Negative, and Potentially-Pathogenic phenotypes in the AMD group were significantly higher than those in the control group. However, the Gram-Positive phenotype had a lower abundance in the AMD group than in the control group. For the Stress-Tolerant phenotype, the abundances of *Escherichia-Shigella*, *Klebsiella*, and *Kluyvera* were significantly higher in the AMD group compared to those in the control group (Figure 6A). For the Gram-Negative phenotype, the abundances of *Escherichia-Shigella*, *Klebsiella*, *Bacteroides*, *Kluyvera*, and *Phascolarctobacterium* were much higher in the AMD group than those in the control group (Figure 6B). For the Potentially-Pathogenic phnotype, the abundances of *Escherichia-Shigella* and *Ruminococcus* were significantly higher in the AMD group than those in the control group. In the AMD group, *Subdoligranulum* was found at a relatively lower abundance than that in the control group (Figure 6C). For the Gram-Positive phenotype, the abundance of *Blautia* was significantly lower in the AMD group than that in the control group and the abundance of *Bifidobacterium* was higher in the AMD group than that in the control group (Figure 6D).

**Figure 7:**
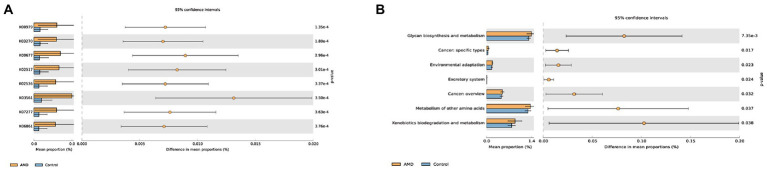
Comparison of the changes in the predicted function abundance at **(A)** KO and **(B)** KEGG level2 pathway between AMD patients and healthy controls. K00979 represents the KdsB; K03270 represents the KdsC; K00677 represents the LpxA; K02517 represents the LpxL; K02536 represents the LpxD; K03561 represents the ExbB; K07277 represents the bamA; and K06861 represents the LptB.

We further predicted KOs and pathway processes of intestinal microbiota using PICRUSt2. A total of 6,507 KOs were found, and the top eight KOs of intestinal microbiota enriched in the AMD group compared with the control group were identified (Supplementary Table 1; Figure 7A). Each K number represents a class of proteins or enzymes with similar sequences and functions in organisms. There were 45 pathways with notable differences between the two groups that were screened out (Supplementary Table 2); the top most distinct pathways are shown in Figure 7B. KEGG level 2 revealed seven metabolic pathways of intestinal microbiota enriched in the AMD group compared with the healthy group, mainly including glycan biosynthesis and metabolism, cancer: specific types, environmental adaptation, excretory system, cancer: overview, metabolism of other amino acids, and xenobiotics biodegradation and metabolism.

## Discussion

The intestinal microbiota is involved in human disease resistance. Since the concept of the gut-retina axis was put forward, the role of intestinal microbiota in regulating eye diseases has been gradually acknowledged (Rowan and Taylor, 2018). Recent studies have revealed the changes and potential applications of gut microbiota in patients with various retinal disorders (Gong et al., 2020; Skondra et al., 2020). A few studies have indicated an association between gut microbiota and AMD (Zinkernagel et al., 2017; Zysset-Burri et al., 2020). To understand this association, we used 16S rRNA gene sequencing in the present study to identify differences in the gut microbiota of patients with AMD and healthy individuals. We found that AMD patients had a different composition of gut microbiota than healthy controls. Moreover, we performed functional predictions of intestinal microbiota in patients with AMD.

In the present study, we found a significant difference in the gut microbiota and specific bacterial taxa between the AMD and control groups. At the phylum level, the AMD group was found to have significantly lower proportions of *Firmicutes*, while the proportions of *Proteobacteria* and *Bacteroidota* were found to be markedly increased. At the genus level, the abundance of *Escherichia-Shigella* increased notably while the abundance of *Blautia* and *Anaerostipes* decreased greatly in the AMD group compared with those in the control group. Enterotype analysis further revealed that *Escherichia-Shigella* was the main microbiota component in the AMD group, and *Blautia* was the main one in the control group. Studies of the gut microbiota to date have found that the composition of the gut microbiota differs between AMD patients and healthy individuals. Zinkernagel et al. found that *Anaerotruncus*, *Oscillibacter*, *Ruminococcus torques*, and *Eubacterium ventriosum* were enriched in AMD patients, and, conversely, *Bacteroides eggerthii* was enriched in control individuals (Zinkernagel et al., 2017). Another larger investigation of human intestinal microbiota found that AMD patients had a higher level of the class *Negativicutes*, whereas healthy controls had a higher level of the genus *Oscillibacter* and species *Bacteroides* (Zysset-Burri et al., 2020).

*Firmicutes*, a main component of intestinal flora, can produce short-chain fatty acids (SCFAs), especially butyrate, which contribute toward maintaining epithelial integrity, reducing appetite and inflammation, and preventing weight gain associated with the consumption of a high-fat diet (HFD; Blaak et al., 2020). Short-chain fatty acids have been found to regulate intestinal microbiota by reducing pH; preventing excessive growth of pathogens, such as *Escherichia coli*; and stimulating the growth of beneficial bacteria of the phylum *Firmicutes* (Duncan et al., 2009; Ghosh et al., 2011; Zimmer et al., 2012). *Blautia*, a member of the *Firmicutes* phylum, has long been recognized for its function in the treatment of inflammatory and metabolic diseases (Liu et al., 2021). The production of bacteriocins enables *Blautia* to inhibit the colonization of pathogenic bacteria in the gut; this ability has allowed its use in probiotics (Liu et al., 2021). One study found that *Blautia* was more enriched in the feces of healthy dogs who were fed with more higher concentrations of potato fiber (Panasevich et al., 2015). Dietary fiber added to HFD for mice reduced their body weight and greatly increased the abundance of *Blautia* in their feces (Yang et al., 2016). A Japanese study revealed that *Blautia* was the only genus with a significant negative correlation between abundance and visceral fat accumulation (Ozato et al., 2019). *Bacteroidota* is composed of various genera with broad metabolic potential, and changes in the abundances of these genera are negatively or positively correlated with human health (Pittayanon et al., 2019). *Proteobacteria* are gram-negative bacteria, and their outer membranes are mainly composed of LPS. Many common human pathogens are found in the *Proteobacteria* phylum, such as *Escherichia-Shigella*, which was found to be associated with increased intestinal permeability and was detected in a variety of disorders (Pedersen et al., 2018). Moreover, increased microbiota instability combined with a rise in the quantities of potential pathogens may affect human hosts by raising the likelihood of local or systemic infections. This kind of instability may lead to increased LPS secretion, which might exacerbate metabolic disorders, creating a vicious loop (Frost et al., 2021). Yang et al. showed that *Anaerostipes* can produce butyric acid, promote the absorption of carbohydrates, and increase the energy utilization efficiency and may thus alleviate the symptoms of irritable bowel syndrome with diarrhea (Yang et al., 2021).

Through phenotypic prediction, we found that microbial phenotypes were different between the AMD and control groups. *Escherichia-Shigella*, *Klebsiella*, *Kluyvera*, *Bacteroides*, *Phascolarctobacterium*, *Ruminococcus*, and *Bifidobacterium* were obviously more enriched in AMD patients, and *Blautia* and *Subdoligranulum* were mainly enriched in the control group. *Klebsiella pneumoniae* is the main pathogen involved in Gram-Negative bacteremia and the most common cause of healthcare-associated infections overall. The gastrointestinal tract is a major reservoir for *Klebsiella pneumoniae* bacteremia (Diekema et al., 2019). *Kluyvera* is a genus of the family Enterobacteriaceae, which caused many kinds of infectious diseases. Many species of this genus have been reported to cause human infection, and *Kluyvera ascorbata* is the most common species causing infection in humans (Lee et al., 2019). A large meta-analysis found a higher abundance of *Proteobacteria*, *Bifidobacterium*, and *Phascolarctobacterium* in patients with Alzheimer’s disease compared with healthy individuals (Hung et al., 2022). A strong correlation exists between Crohn’s disease (CD) and *Ruminococcus gnavus*. Some researchers found that an increased relative abundance of *Ruminococcus gnavus* in the gut microbiota was associated with an increase in CD and various other inflammatory diseases (Hall et al., 2017; Nishino et al., 2018). Another research found that *Ruminococcus* was independently associated with liver fibrosis (Boursier et al., 2016). Upon examining *Subdoligranulum* in two cohorts of overweight/obese individuals (from two separate studies), researchers found that the abundance of *Subdoligranulum* had a positive correlation with the HDL-cholesterol level and a negative correlation with a variety of metabolic risks parameters (Van Hul et al., 2020). Another study revealed that *Subdoligranulum* improved necrotizing enterocolitis by producing butyrate to regulate gut function (Lin et al., 2021).

A predictive function analysis was next used to better investigate the rich metabolic activities of the gut microbiota. K00979 represents KdsB, also known as CMP-KDO synthetase or KDO cytidylyltransferase. In Gram-Negative bacteria, KdsB is an important enzyme in the biosynthesis of LPS. K03270 represents KdsC, which is also a key enzyme in the synthesis of KDO. Any interruption in the KDO biosynthesis pathway can reduce the pathogenicity of bacteria (Ahmad et al., 2019). Lipid A is a highly conserved component of LPS. Nine essential enzymes, including K00677, K02517, and K02536, were involved in the Lipid A biosynthesis pathway (Emiola et al., 2014). K03561 represents the biopolymer transport protein ExbB. TonB complex, composed of TonB, ExbB, and ExbD, is an energy provider for the active transport of external nutrients by bacteria. A great number of studies have shown that the TonB system is not only involved in nutrient transport but also in bacterial signal transduction and, indirectly, in bacterial virulence (Porcheron et al., 2013). K07277 represents the outer membrane protein insertion porin family or bamA. BamA is the core component of the β⁃barrel assembly machinery (BAM) complex, which plays a key role in the immunogenicity, pathogenicity, and multidrug resistance of bacteria (Noinaj et al., 2013; Babu et al., 2018). K06861 represents the LPS export system ATP-binding protein or LptB. The absence of any Lpt component prevents LPS transport to the outer membrane of gram-negative bacteria (Bowyer et al., 2011). In the present study, KEGG analysis revealed the top seven pathways linked to the abovementioned gut microbiota that were altered in the AMD group. These pathways were also reported to be involved in inflammatory disease in other studies. *E. ramulus*, of the *Firmicutes* phylum, has been found to cleave quercetin and produce 3,4-dihydroxyphenylacetate, which inhibits colon cancer cell proliferation (Gao et al., 2006). Moreover, *Bacteroides thetaiotaomicron*, of the *Bacteroidota* phylum, is thought to be able to metabolize complex substrates, like mucin; it is thus important to learn whether it contributes to inflammation and infection by weakening the mucosal barrier (Tramontano et al., 2018).

However, there are several limitations to this research as well. First, since this was single-center research with a small sample size, further multi-center studies are required to expand the sample size. Second, our research predicted metabolic functions of gut microbiota in AMD patients and healthy controls; further analysis of bacterial metabolites is needed to determine their potential underlying mechanisms. Third, we found that the proportions of *Blautia* and *Anaerostipes* in the AMD group were lower than those in the control group. Contrarily, the abundances of *Escherichia-Shigella*, *Bacteroides*, and *Bifidobacterium* in the AMD group were higher than those in the control group. Whether to change the abundances of these bacteria in the guts of patients with AMD or to use the metabolites produced by these bacteria to treat AMD remains to be further explored.

In conclusion, the present study revealed alterations in the compositions and metabolic functions of intestinal microbiota between AMD patients and healthy individuals. Moreover, it proposed specific bacteria and potential microbial markers-that can be targeted for AMD treatment. These findings clarify the mechanism underlying AMD from the perspective of intestinal microbial dysbiosis. In addition, the findings provide a foundation prospect for improving the balance of the intestinal microenvironment to treat AMD.

## Data availability statement

The data presented in the study are deposited in the SRA database repository accession number PRJNA799475.

## Ethics statement

The studies involving human participants were reviewed and approved by The ethics committee of Shanghai Tenth People’s Hospital. The patients/participants provided their written informed consent to participate in this study.

## Author contributions

QP, MW, and RD conceived and designed the study. YZ wrote the manuscript. YZ, TW, and ZW performed the experiments. JB and YX analyzed the data. YZ, TW, YX, JB, and ZW reviewed the manuscript. All authors contributed to the article and approved the submitted version.

## Funding

This work was supported by the National Natural Science Foundation of China (grant numbers 62271337 and 81470029) and Three-Year Action Plan for Promoting Clinical Skills and Clinical Innovation in Municipal Hospitals (grant number SHDC2020CR5014).

## Conflict of interest

The authors declare that the research was conducted in the absence of any commercial or financial relationships that could be construed as a potential conflict of interest.

## Publisher’s note

All claims expressed in this article are solely those of the authors and do not necessarily represent those of their affiliated organizations, or those of the publisher, the editors and the reviewers. Any product that may be evaluated in this article, or claim that may be made by its manufacturer, is not guaranteed or endorsed by the publisher.

## Footnotes

^1^https://ccb.jhu.edu/software/FLASH/index.shtml

^2^http://rdp.cme.msu.edu/

^3^https://bugbase.cs.umn.edu/index.html

^4^https://github.com/picrust/picrust2/
