# Supplementary figures and images for: Alterations of the intestinal microbiota in age-related macular degeneration
Source: Front Microbiol. 2023 Apr 5;14:1069325. doi: 10.3389/fmicb.2023.1069325 (PMC10113553; doi:10.3389/fmicb.2023.1069325)

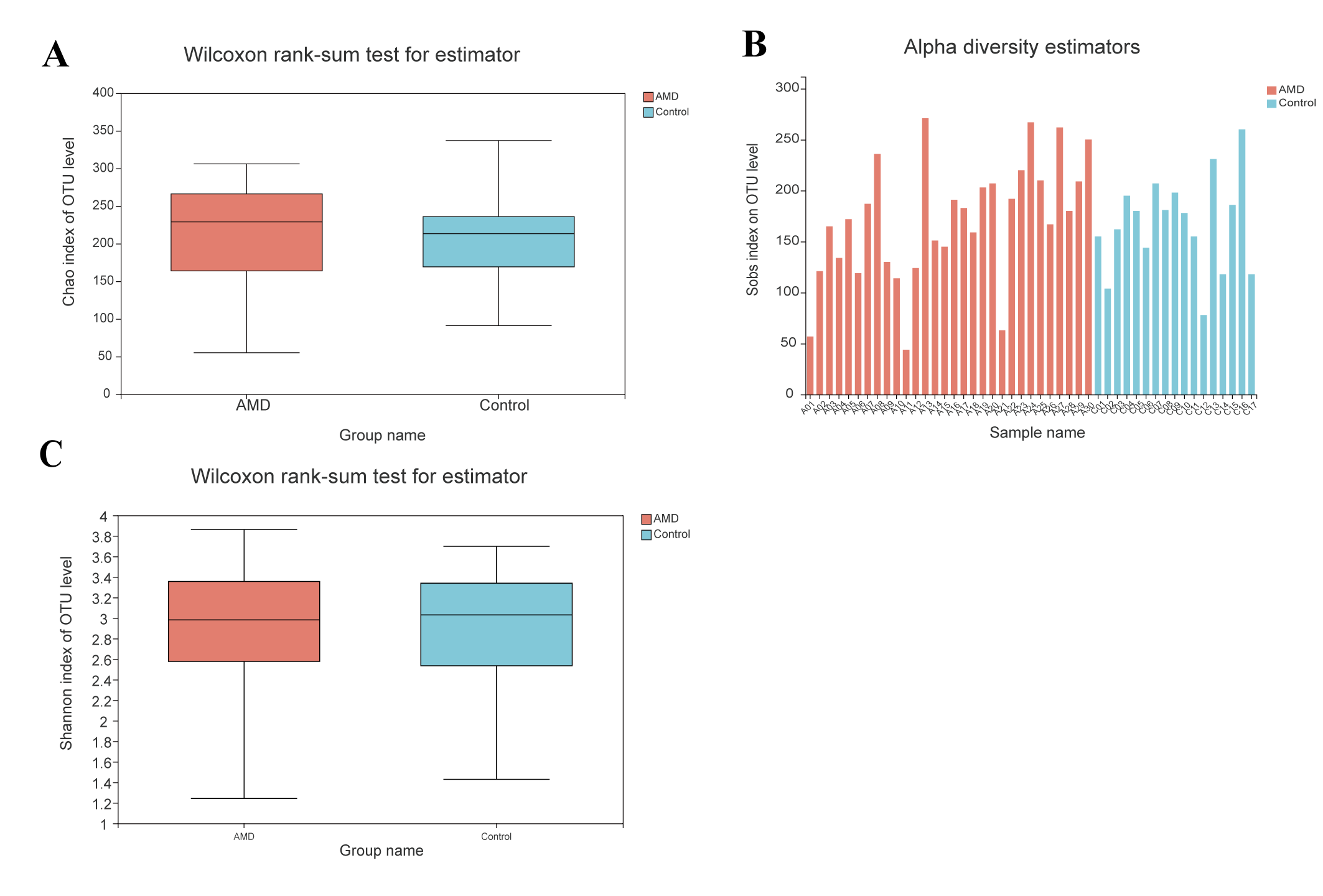

Supplement: Supplementary file 3 [file Image_1_v1.TIF]

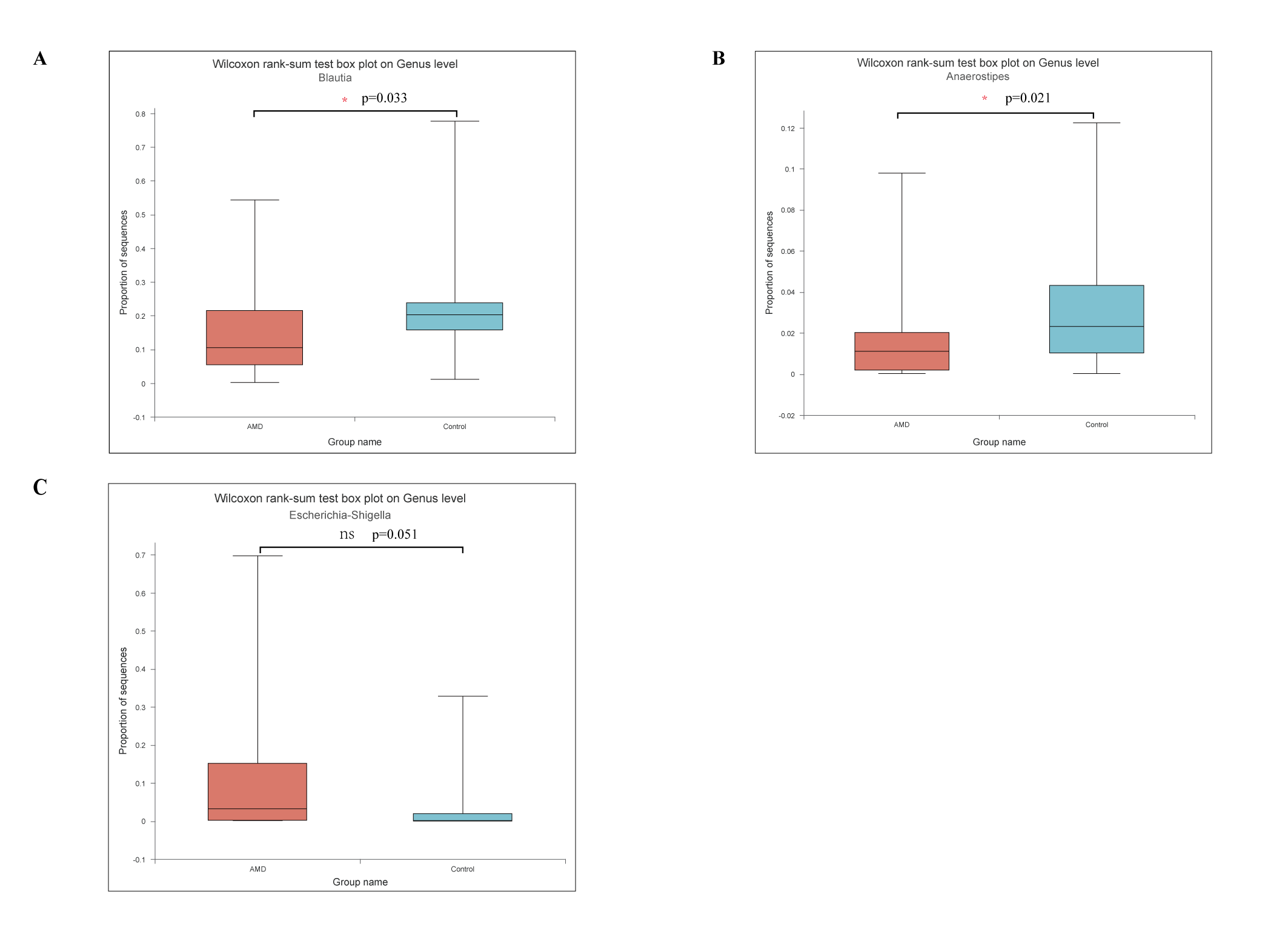

Supplement: Supplementary file 4 [file Image_2_v1.TIF]
